# Supplementary material for: Canonical versus non-canonical transsynaptic signaling of neuroligin 3 tunes development of sociality in mice
Source: Nat Commun. 2021 Mar 23;12:1848. doi: 10.1038/s41467-021-22059-6 (PMC7988105; doi:10.1038/s41467-021-22059-6)
Supplement: Supplementary file 1 — Supplementary Information [file 41467_2021_22059_MOESM1_ESM.pdf]

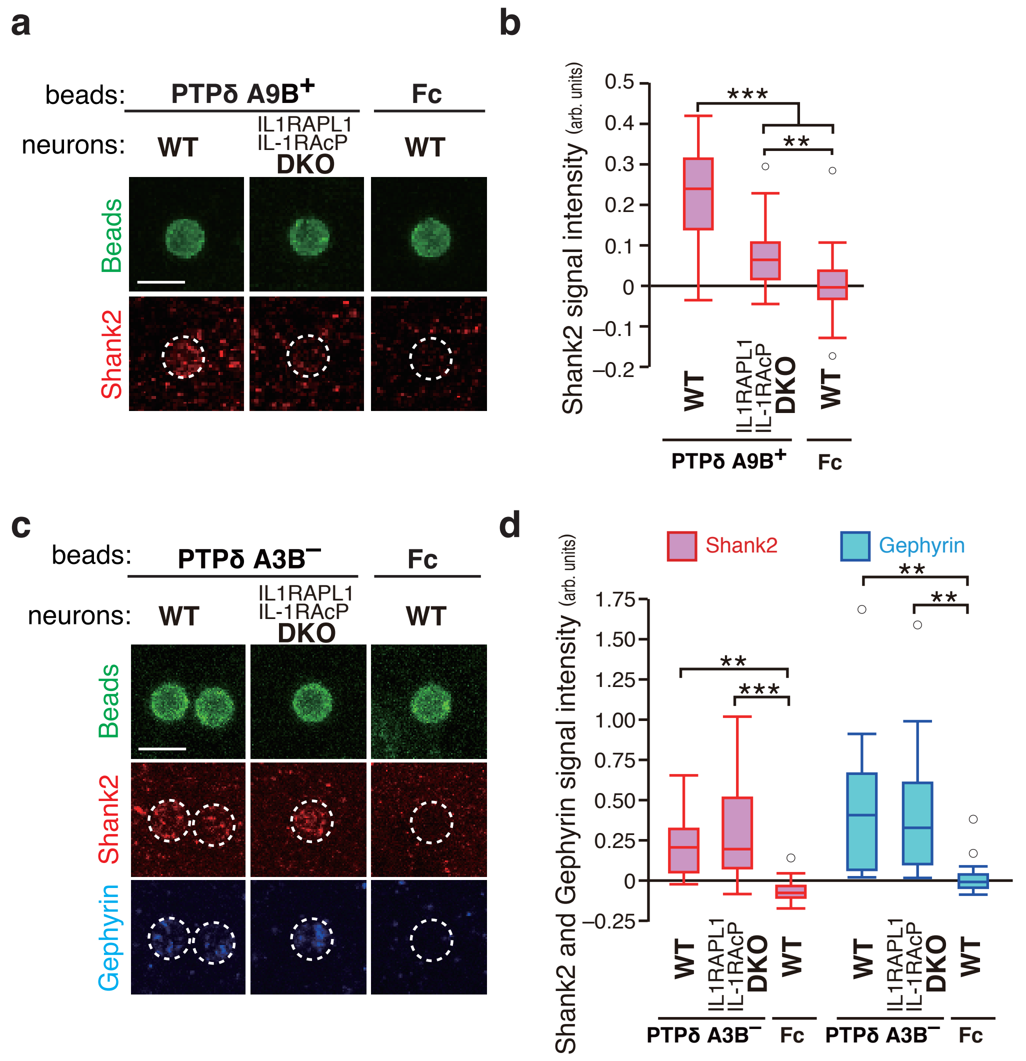

Supplementary Fig. 1: Effects of IL1RAPL1 and IL-1RAcP double knockout on postsynaptic differentiation induced by PTPδ splice variants.

a, Induction of excitatory postsynaptic differentiation of cerebral cortical neurons from wild-type and IL1RAPL1/IL-1RAcP double knockout (DKO) mice by magnetic beads coated with the ECD of meB -containing PTPδ variant (PTPδA9B^{+}) was visualized by immunostaining for Shank2 (red). **b**, Intensity of staining signals for Shank2 in **a** (n = 36 and 48 PTPδA9B^{+} beads for WT an DKO, respectively; n = 45 Fc beads). **c**, Induction of excitatory and inhibitory postsynaptic differentiation of cerebral cortical neurons from wild-type and IL1RAPL1/IL-1RAcP DKO mice by beads conjugated with the ECD of meB-lacking PTPδ variant (PTPδA3B^{-}) was visualized by immunostaining for Shank2 (red) and gephyrin (blue). **d**, Intensity of staining signals for Shank2 (red bars) and gephyrin (blue bars) in **c** (n = 18 PTPδA3B^{-} beads each for WT and DKO; n = 16 Fc beads). Scale bars represent 5 µm. Data are presented as box plots. Horizontal line in each box shows median, box shows the IQR and the whiskers are 1.5 × IQR. **$P < 0.01$ and *** $p < 0.001$, Tukey' s post hoc test. See Supplementary Table 4 for additional statistics and exact p values.

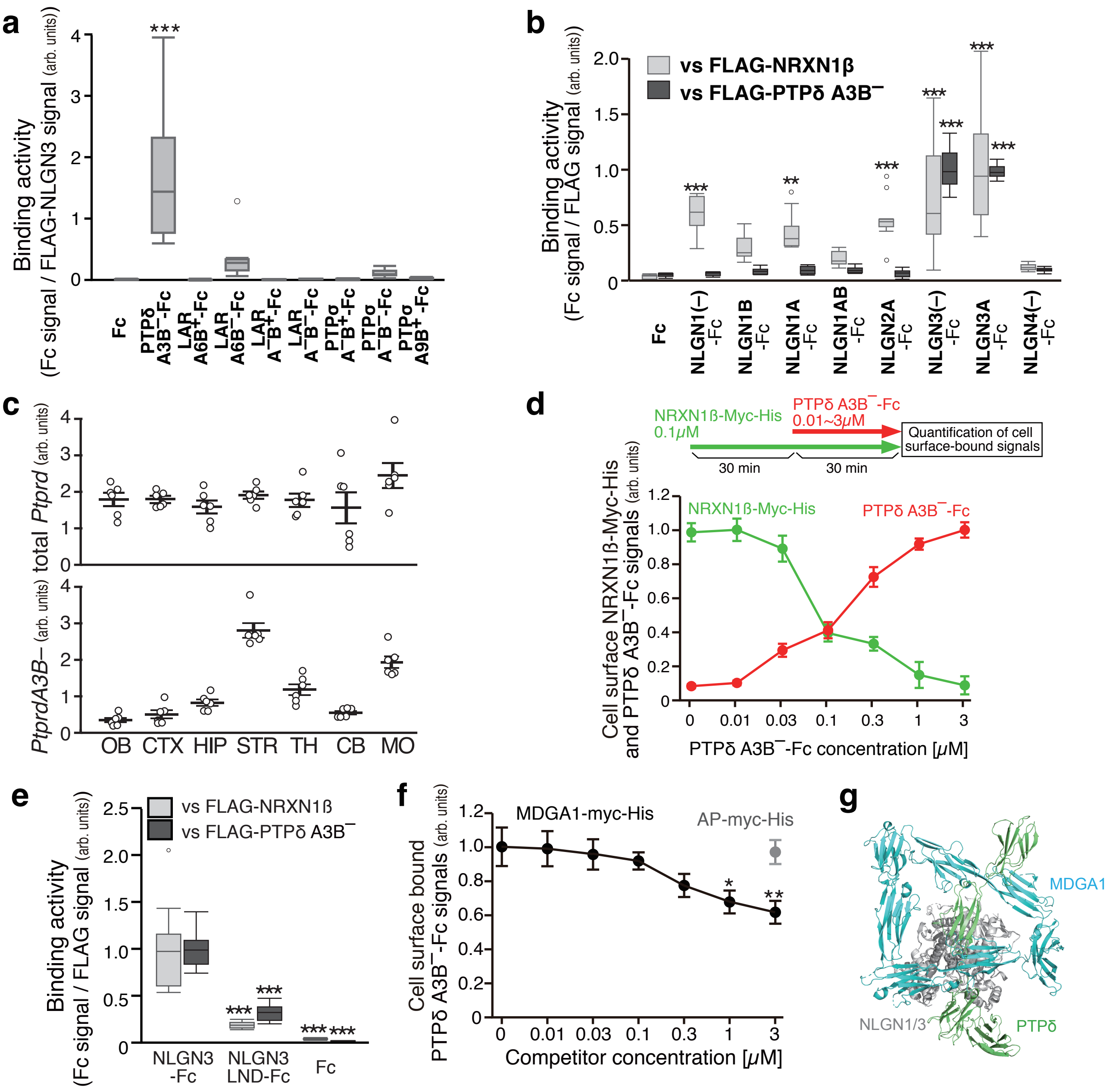

Supplementary Fig. 2: Selective interaction between NLGN3 and PTPδ splice variants lacking meB. **a**, Ratios of cell surface staining signals for ECDs of LAR and PTPσ splice variants fused to Fc and FLAG-NLGN3 expressed on HEK293T cells (n = 15 cells each). **b**, Ratios of cell surface staining signals for Fc fusion proteins of ECDs of the NLGN family and FLAG-NRXN1β (gray bars) or FLAG-PTPδA3B⁻ (black bars) expressed on HEK293T cells (n = 12 cells each). **c**, Brain regional variation of total *Ptprd* and *Ptprd*A3B⁻ variant expression. Total *Ptprd* (top) and *Ptprd*A3B⁻ variant (bottom) expression levels estimated by real-time PCR are shown (n = 6 each, triplicate experiments for 2 mice). OB, olfactory bulb; CTX, cerebral cortex; HIP, hippocampus; STR, striatum; TH, thalamus; CB, cerebellum; MO, medulla oblongata. **d**, After NLGN3-NRXN1β complexes were formed on HEK293T cells expressing FLAG-NLGN3, PTPδA3B⁻-Fc was added at the concentrations of 0–3.0 µM. Cell surface bound signals for NRXN1β-Myc-His and PTPδA3B⁻-Fc were quantified (n = 20 cells each). **e**, Ratios of cell surface staining signals for ECDs of wild-type or L374A/N375A/D377A mutated form (LND) of NLGN3 fused to Fc and FLAG-NRXN1β or FLAG-PTPδA3B⁻ expressed on HEK293T cells (n = 10 cells each). **f**, Cell surface bound PTPδA3B⁻-Fc signals on FLAG-NLGN3-expressing HEK293T cells in the presence of 0–3.0 µM MDGA1-Myc-His were quantified (n = 20 cells each). **g**, Superposition of the MDGA1-bound NLGN1 (PDB 5OJR) onto the PTPδA3B⁻-bound NLGN3. The interface between PTPδA3B⁻ and NLGN3 is overlapped with that between MDGA1 and NLGN1. Data in **a**, **b**, and **e** are presented as box plots. Horizontal line in each box shows median, box shows the IQR and the whiskers are 1.5 × IQR. Values in **d** and **f** represent mean ± s.e.m. *$P < 0.05$, **$P < 0.01$ and ***$P < 0.001$, Two-sided Dunnett's test in **a**, **b** and **f**, and Tukey's post hoc test in **e**.
See Supplementary Table 4 for additional statistics and exact p values.

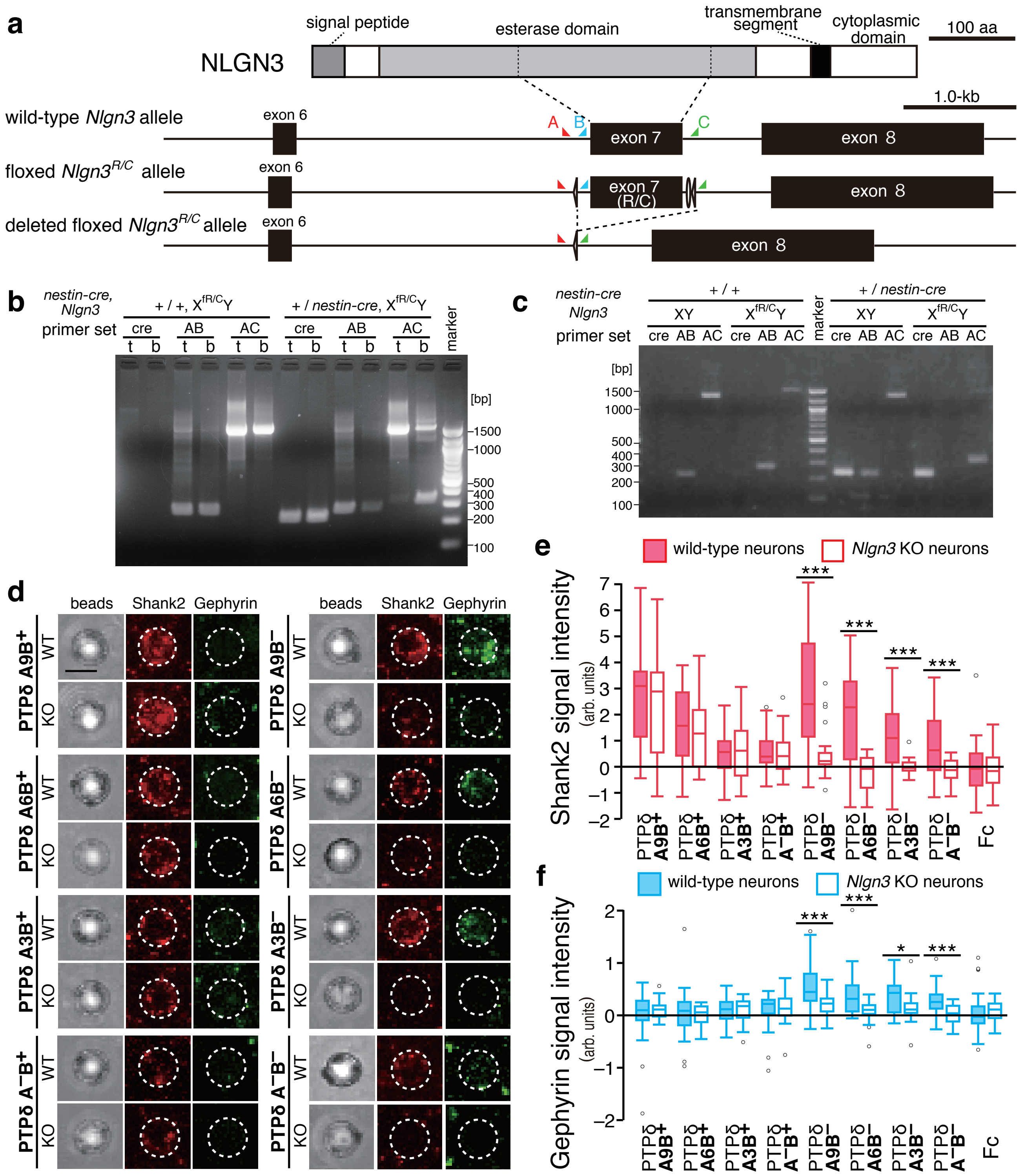

Supplementary Fig. 3: PTPδ splice variants lacking meB require NLGN3 for postsynaptic differentiation. **a**, Schema for generation of neuron-specific *Nlgn3* exon 7-deleted mice. *Nlgn3* exon 7-deleted mice were generated by crossing heterozygous female $XX^{fR/C}$ mice, which carry floxed exon 7 with a point mutation corresponding to the human NLGN3 R451C, with nestin-Cre transgenic male mice (+/nestin-cre) carrying the cre recombinase gene under the control of the rat nestin promoter and enhancer. **b**, Detection of undeleted and deleted floxed Nlgn3 alleles by PCR using primers A, B, and C against genomic DNA from tails (t) and brains (b) of adult male $X^{fR/C}Y$ mice with or without the nestin-Cre transgene. **c**, PCR detection of *Nlgn3* exon 7 deletion in DIV8 cortical neurons prepared from $X^{fR/C}Y$, +/nestin-cre mice using primers A, B, and C. **d**, Induction of postsynaptic differentiation of wild-type and NLGN3 knockout cerebral cortical neurons by magnetic beads coated with ECDs of PTPδ splice variants fused to Fc. Excitatory and inhibitory postsynaptic terminals induced by the beads were visualized by immunostaining for Shank2 (red) and gephyrin (green), respectively. **e** and **f**, Intensity of staining signals for Shank2 (**e**) and gephyrin (**f**) on the beads was quantified (n = 24 and 29 PTPδA9B^{+}-beads, 27 and 26 PTPδA6B^{+}-beads, 33 and 28 PTPδA3B^{+}-beads, 27 and 26 PTPδA^{-}B^{+}-beads, 30 and 31 PTPδA9B^{-}-beads, 24 and 30 PTPδA6B^{-}-beads, 24 and 28 PTPδA3B^{-}-beads, 34 and 31 PTPδA^{-}B^{-}-beads, and 28 and 32 Fc-beads, for wild -type and Nlgn3 KO neurons, respectively). Data are presented as box plots. Horizontal line in each box shows median, box shows the IQR and the whiskers are 1.5 × IQR. *p < 0.05 and ***p < 0.001, Two-sided Student' s t-test. Scale bar represents 5 µm. See Supplementary Table 4 for additional statistics and exact p values.

a

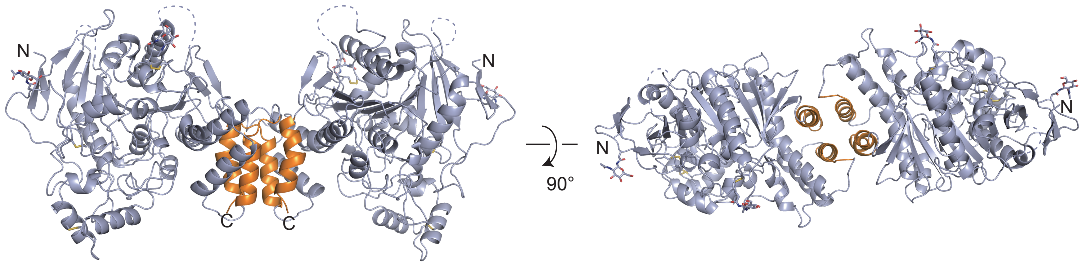

b

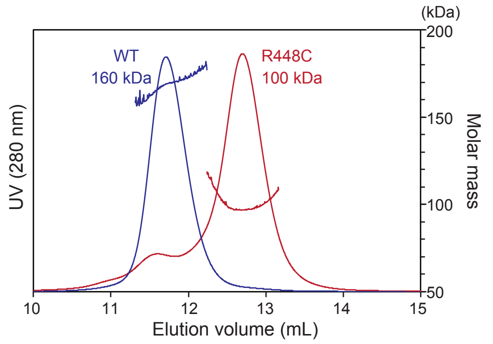

c

```
mNLGN3      254 GDPRRITVFGSGIGASCVSLLTLSHHSEGLFQRAI---------IQSGSALSSWAVNYQP 304
rNLGN3      277 GDPRRITVFGSGIGASCVSLLTLSHHSEGLFQRAI---------IQSGSALSSWAVNYQP 327
hNLGN3      277 GDPRRITVFGSGIGASCVSLLTLSHHSEGLFQRAI---------IQSGSALSSWAVNYQP 327
mNLGN1      270 GDPLRITVFGSGAGGSCVNLLTLSHYSEGNRWSNSTKGLFQRAIAQSGTALSSWAVSFQP 329
rNLGN1      270 GDPLRITVFGSGAGGSCVNLLTLSHYSEGNRWSNSTKGLFQRAIAQSGTALSSWAVSFQP 329
hNLGN1      267 GDPLRITVFGSGAGGSCVNLLTLSHYSEGNRWSNSTKGLFQRAIAQSGTALSSWAVSFQP 326
mNLGN2      254 GDPERITIFGSGAGASCVNLLILSHHSEGLFQKAI---------AQSGTAISSWSVNYQP 304
rNLGN2      254 GDPERITIFGSGAGASCVNLLILSHHSEGLFQKAI---------AQSGTAISSWSVNYQP 304
hNLGN2      254 GDPERITIFGSGAGASCVNLLILSHHSEGLFQKAI---------AQSGTAISSWSVNYQP 304
mNLGN4      252 GDPDRVTVFGSGAGASCVSLLTLSHYSEGLFQKAI---------IQSGTALSSWAVNYQP 302
hNLGN4-X    243 GDPKRVTIFGSGAGASCVSLLTLSHYSEGLFQKAI---------IQSGTALSSWAVNYQP 293
hNLGN4-Y    243 GDPKRVTIFGSGAGASCVSLLTLSHYSEGLFQKAI---------IQSGTALSSWAVNYQP 293

mNLGN3      305 VKYTSLLADKVGCN------------VLDTVDMVDCLRQKSAKELVEQDIQPARYHVAFG 352
rNLGN3      328 VKYTSLLADKVGCN------------VLDTVDMVDCLRQKSAKELVEQDIQPARYHVAFG 375
hNLGN3      328 VKYTSLLADKVGCN------------VLDTVDMVDCLRQKSAKELVEQDIQPARYHVAFG 375
mNLGN1      330 AKYARILATKVGCN------------VSDTVELVECLQKKPYKELVDQDVQPARYHIAFG 377
rNLGN1      330 AKYARILATKVGCN------------VSDTVELVECLQKKPYKELVDQDVQPARYHIAFG 377
hNLGN1      327 AKYARMLATKVGCN------------VSDTVELVECLQKKPYKELVDQDIQPARYHIAFG 374
mNLGN2      305 LKYTRLLAAKVGCD------------REDSTEAVECLRRKSSRELVDQDVQPARYHIAFG 352
rNLGN2      305 LKYTRLLAAKVGCD------------REDSTEAVECLRRKSSRELVDQDVQPARYHIAFG 352
hNLGN2      305 LKYTRLLAAKVGCD------------REDSAEAVECLRRKPSRELVDQDVQPARYHIAFG 352
mNLGN4      303 ARYARALGERVGCATPDPGSPPGSPPGWDSASLVSCLRGKAAGELARARVTPATYHVAFG 362
hNLGN4-X    294 AKYTRILADKVGCN------------MLDTTDMVECLKNKNYKELIQQTITPATYHIAFG 341
hNLGN4-Y    294 AKYTRILADKVGCN------------MLDTTDMVECLRNKNYKELIQQTITPATYHIAFG 341

mNLGN3      353 PVIDGDVIPDDPEILMEQGEFLNYDIMLGVNQGEGLKFVEG-VVDPEDGVSGTDFDYSVS 411
rNLGN3      376 PVIDGDVIPDDPEILMEQGEFLNYDIMLGVNQGEGLKFVEG-VVDPEDGVSGTDFDYSVS 434
hNLGN3      376 PVIDGDVIPDDPEILMEQGEFLNYDIMLGVNQGEGLKFVEG-VVDPEDGVSGTDFDYSVS 434
mNLGN1      378 PVIDGDVIPDDPQILMEQGEFLNYDIMLGVNQGEGLKFVEN-IVDSDDGVSASDFDFAVS 436
rNLGN1      378 PVIDGDVIPDDPQILMEQGEFLNYDIMLGVNQGEGLKFVEN-IVDSDDGVSASDFDFAVS 436
hNLGN1      375 PVIDGDVIPDDPQILMEQGEFLNYDIMLGVNQGEGLKFVEN-IVDSDDGISASDFDFAVS 433
mNLGN2      353 PVVDGDVVPDDPEILMQQGEFLNYDMLIGVNQGEGLKFVED-SAESEDGVSASAFDFTVS 411
rNLGN2      353 PVVDGDVVPDDPEILMQQGEFLNYDMLIGVNQGEGLKFVED-SAESEDGVSASAFDFTVS 411
hNLGN2      353 PVVDGDVVPDDPEILMQQGEFLNYDMLIGVNQGEGLKFVED-SAESEDGVSASAFDFTVS 411
mNLGN4      363 PTVDGDVIPDDPQILMEQGEFLNYDIMLGVNQGEGARFVDGLGGGHDGGYGGYGGGYGGG 422
hNLGN4-X    342 PVIDGDVIPDDPQILMEQGEFLNYDIMLGVNQGEGLKFVDG-IVDNEDGVTPNDFDFSVS 400
hNLGN4-Y    342 PVIDGDVIPDDPQILMEQGEFLNYDIMLGVNQGEGLKFVDG-IVDNEDGVTPNDFDFSVS 400

mNLGN3      601 WKHLVPHLYNLH----DMFHYTST---TTKVPPPDTTHSS--------HITRRPNGKTWS 645
rNLGN3      624 WKHLVPHLYNLH----DMFHYTST---TTKVPPPDTTHSS--------HITRRPNGKTWS 668
hNLGN3      624 WKHLVPHLYNLH----DMFHYTST---TTKVPPPDTTHSS--------HITRRPNGKTWS 668
mNLGN1      626 WLELVPHLHNLN----DISQYT------------STTTKV--------PSTDITLRPTRK 661
rNLGN1      626 WLELVPHLHNLN----DISQYT------------STTTKV--------PSTDITLRPTRK 661
hNLGN1      623 WLELVPHLHNLN----DISQYT------------STTTKV--------PSTDITFRPTRK 658
mNLGN2      601 WLELVPHLHNLH----TELFTT-----TTRLPPYATRWPP--------RTPGPGTSGTRR 643
rNLGN2      601 WLELVPHLHNLH----TELFTT-----TTRLPPYATRWPP--------RTPGPGTSGTRR 643
hNLGN2      601 WLELVPHLHNLH----TELFTT-----TTRLPPYATRWPP--------RPPA-GAPGTRR 642
mNLGN4      663 WLELVPHLHGLAA---DPGAYLSAA--ATRAAPSGDPDRDPGGGGGGRRRPRPATRRPAV 717
hNLGN4-X    590 WLELVPHLHNLNEIFQYVSTTTKVPPPDMTSFPYGTRRSP--------AKIWPTTKRPAI 641
hNLGN4-Y    590 WLELVPHLHNLNEIFQYVSTTTKVPPPDMTSFPYGTRRSP--------AKIWPTTKRPAI 641
```

Supplementary Fig. 4: Structure of the apo-NLGN3 ECD dimer. **a**, Overall structure of the NLGN3 dimer. The coloring scheme is the same as that in Fig. 3a. **b**, SEC-MALS analyses of wild type (blue) and the R448C mutant (red) of mouse NLGN3 ECD. Chromatograms and determined molar masses are shown. **c**, Amino-acid sequence alignment of NLGN1–NLGN4. The PTPδ-interacting residues of mNLGN3, except Gly225, are indicated by red inverted triangles. NLGN3 residues critical for binding to both PTPδ and NRXN, and the corresponding residues of other NLGNs, are highlighted by yellow boxes. NLGN3 residues critical for binding to NRXN but not to PTPδ and the corresponding residues of other NLGNs are highlighted by white letters in brown boxes. m, Mus musculus; r, Rattus norvegicus; h, Homo sapiens.

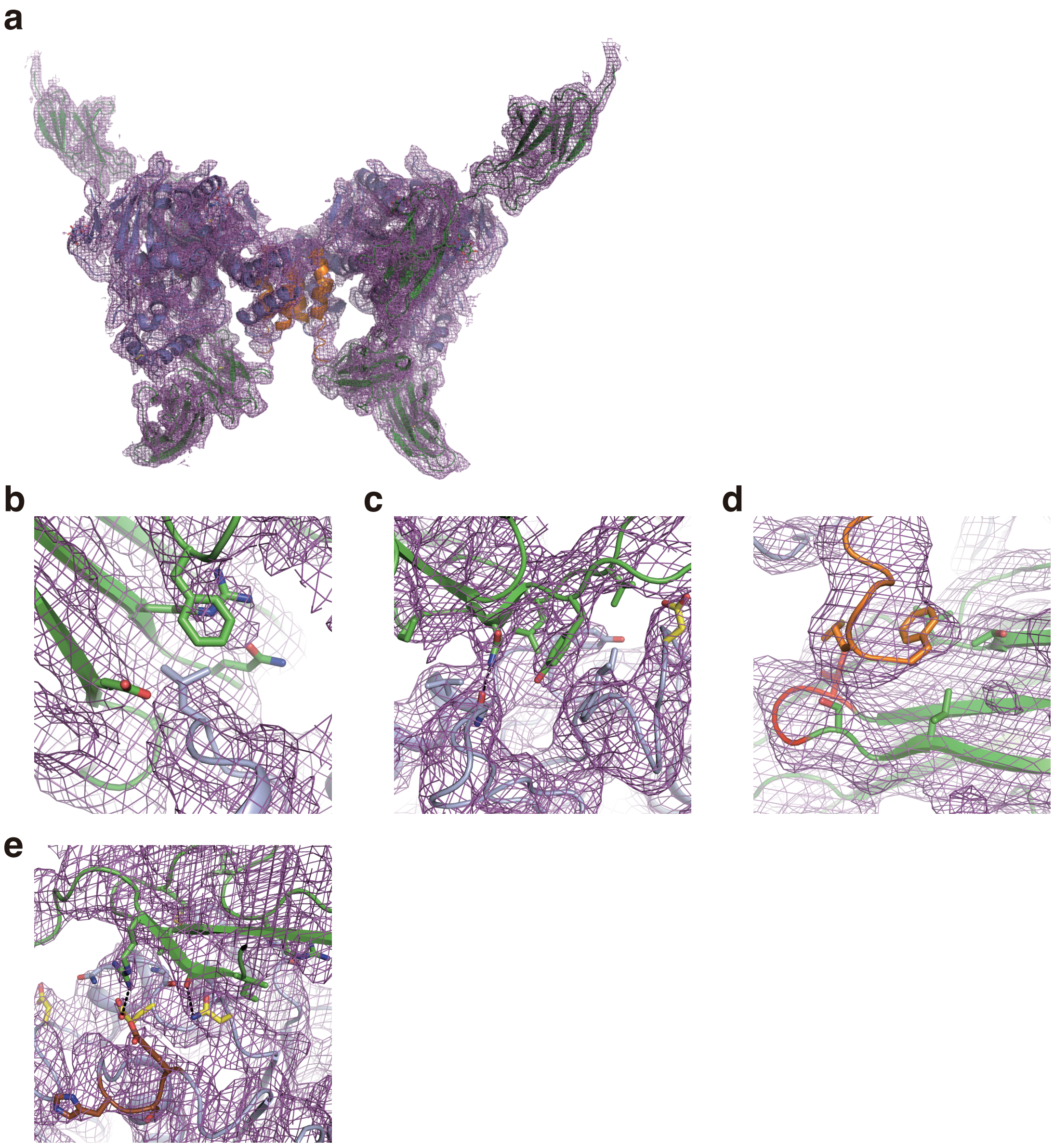

Supplementary Fig. 5: Electron density map of the NLGN3–PTPδA3B^- complex.

a, Overall view covering the dimeric complex, corresponding to Fig. 3a. **b-d**, Close-up views showing the NLGN3 α/β-hydrolase-fold core/PTPδ Ig1 interface (**b**), NLGN3 α/β-hydrolase-fold core/PTPδ Ig2 interface (**c**), and NLGN3 ECD C-terminal/PTPδ Ig2 interface (**d**), corresponding to Fig. 4a,b,c, respectively. **e**, Close-up view showing the NLGN3/PTPδ Ig3 interface, corresponding to Fig. 4d. A 2Fo–Fc map contoured at 1.0 σ level is shown as mesh with the model of the complex.

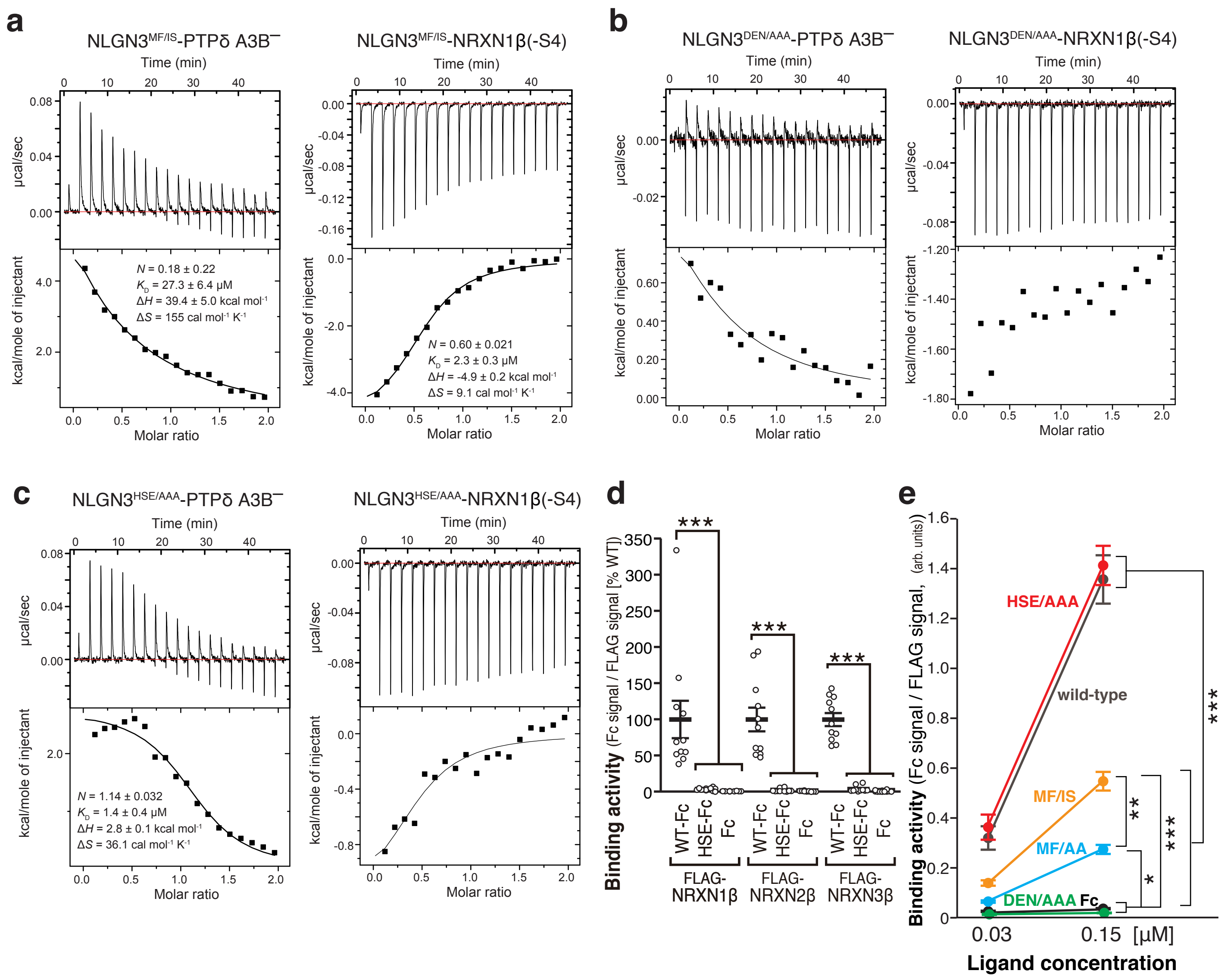

Supplementary Fig. 6: Binding properties of NLGN3 mutants to PTPδA3B⁻ and NRXN1β. **a**, ITC titration of PTPδA3B⁻ Ig1–Fn1 (left) and NRXN1β (right) to NLGN3 MF/IS. **b**, ITC titration of PTPδA3B⁻ Ig1–Fn1 (left) and NRXN1β (right) to NLGN3 DEN/AAA. **c**, ITC titration of PTPδA3B⁻ Ig1–Fn1 (left) and NRXN1β (right) to NLGN3 HSE/AAA. The calculated thermodynamic parameters are also listed in **a** and **c**. The error of K_D was estimated by dividing the error of K_A by K_A^2. **d**, Ratios of cell surface staining signals for ECDs of wild-type NLGN3 and NLGN3 HSE/AAA fused to Fc and FLAG-NRXN1β, FLAG-NRXN2β, and FLAG-NRXN3β expressed on HEK293T cells (n = 11 cells each).**e**, Ratios of cell surface staining signals for mutated forms and wild-type of NLGN3 ECDs fused to Fc and FLAG-PTPδA3B⁻ expressed on HEK293T cells (n = 29, 24, 19, 26, 26, 26 cells for 0.03 μM wild-type, HSE/AAA, MF/IS, MF/AA, DEN/AAA and Fc, respectively; n = 27, 27, 26, 26, 26 and 24 cells for 0.15 μM wild-type, HSE/AAA, MF/IS, MF/AA, DEN/AAA and Fc, respectively). *$p < 0.05$, **$p < 0.01$, and ***$p < 0.001$, Tukey' s post hoc test. Data are mean ± s.e.m. See Supplementary Table 4 for additional statistics and exact p values.

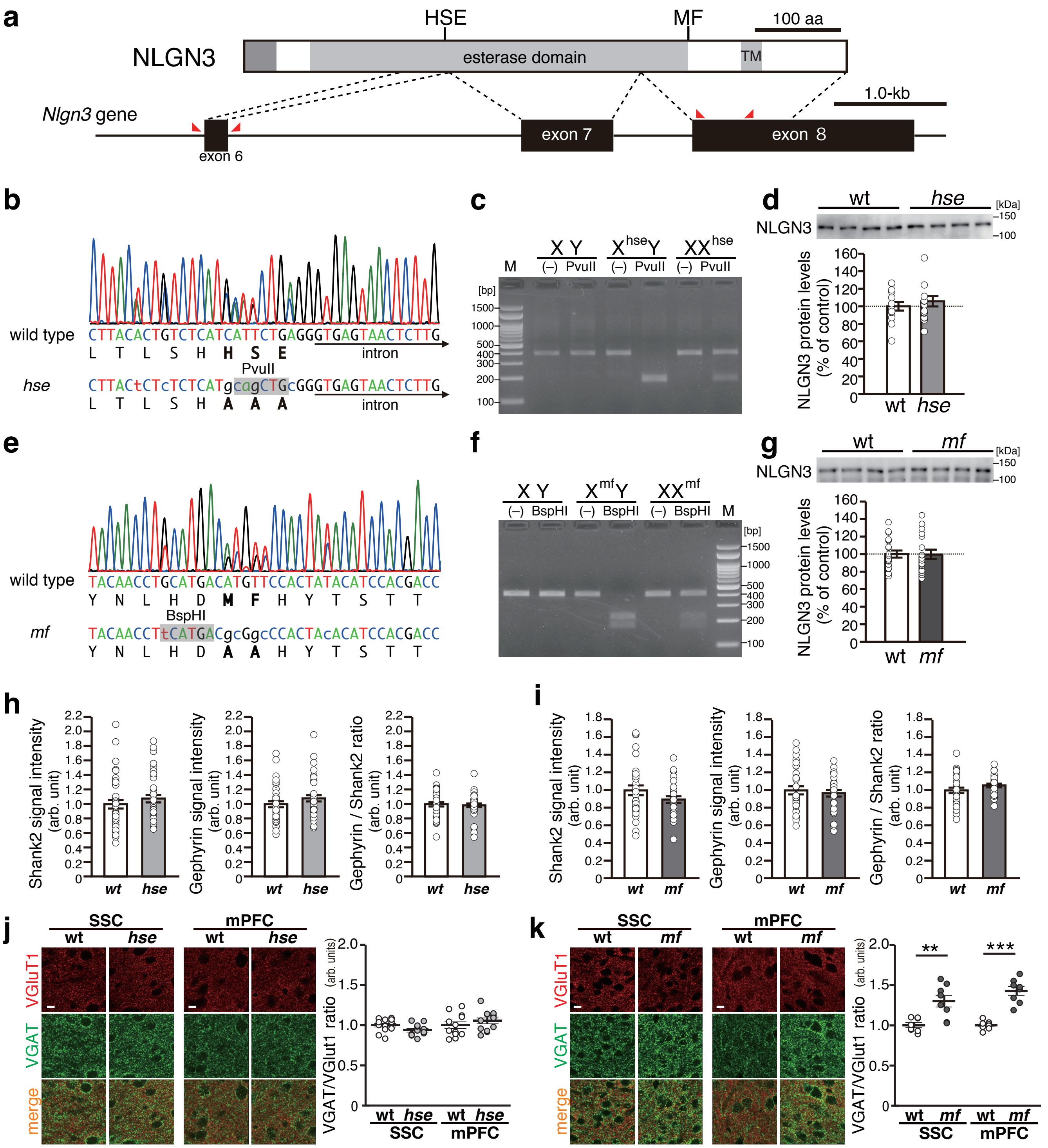

Supplementary Fig. 7: Generation of *Nlgn3^hse^* and *Nlgn3^mf^* mutant mice. **a**, Structures of the NLGN3 protein and *Nlgn3* gene. **b**, Nucleotide sequence of genomic PCR fragment of *Nlgn3* exon 6 from *Nlgn3^hse^* heterozygous female mouse. **c**, Patterns of PvuII-digested PCR fragment of *Nlgn3* exon 6 from wild-type male, *Nlgn3^hse^* male, and *Nlgn3^hse^* heterozygous female mouse. **d**, A representative immunoblot (top) and quantification of expression levels (bottom) of NLGN3 in *Nlgn3^hse^* mutant mice and their wild-type littermates (N = 14 male mice each). **e**, Nucleotide sequence of genomic PCR fragment of *Nlgn3* exon 8 from *Nlgn3^mf^* heterozygous female mouse. **f**, Patterns of BspHI-digested PCR fragment of *Nlgn3* exon 8 from wild-type male, *Nlgn3^mf^* male, and *Nlgn3^mf^* heterozygous female mouse. **g**, A representative immunoblot (top) and quantification of expression levels (bottom) of NLGN3 protein in *Nlgn3^mf^* mutant mice and their wild-type littermates (N = 18 and 20 male mice, respectively). **h** and **i**, Shank2 and Gephyrin immunostaining signal intensity in cultured cortical neurons from *Nlgn3^hse^* (n = 39 optical fields) and their littermate controls (n = 38 optical fields) (**h**) and those from *Nlgn3^mf^* (n = 28 optical fields) and their littermate controls (n = 31 optical fields) (**i**). Gephyrin/Shank2 signal ratios are shown on the right. **j** and **k**, Immunohistochemistry for VGluT1 and VGAT in somatosensory cortex (SSC) and medial prefrontal cortex (mPFC) of *Nlgn3^hse^* (**j**) and *Nlgn3^mf^* (**k**) mutant mice. Ratios of staining signal intensity for VGAT and VGluT1 are quantified on the right (n = 12 slices from 3 mice each for *Nlgn3^hse^* and wild-type control; n = 8 slices from 2 mice each for *Nlgn3^mf^* and wild-type control). Scale bars, 10 µm. Data are mean ± s.e.m. **p < 0.01 and *** p < 0.001, two-sided Mann-Whitney U-test. See Supplementary Table 4 for additional statistics and exact p values.

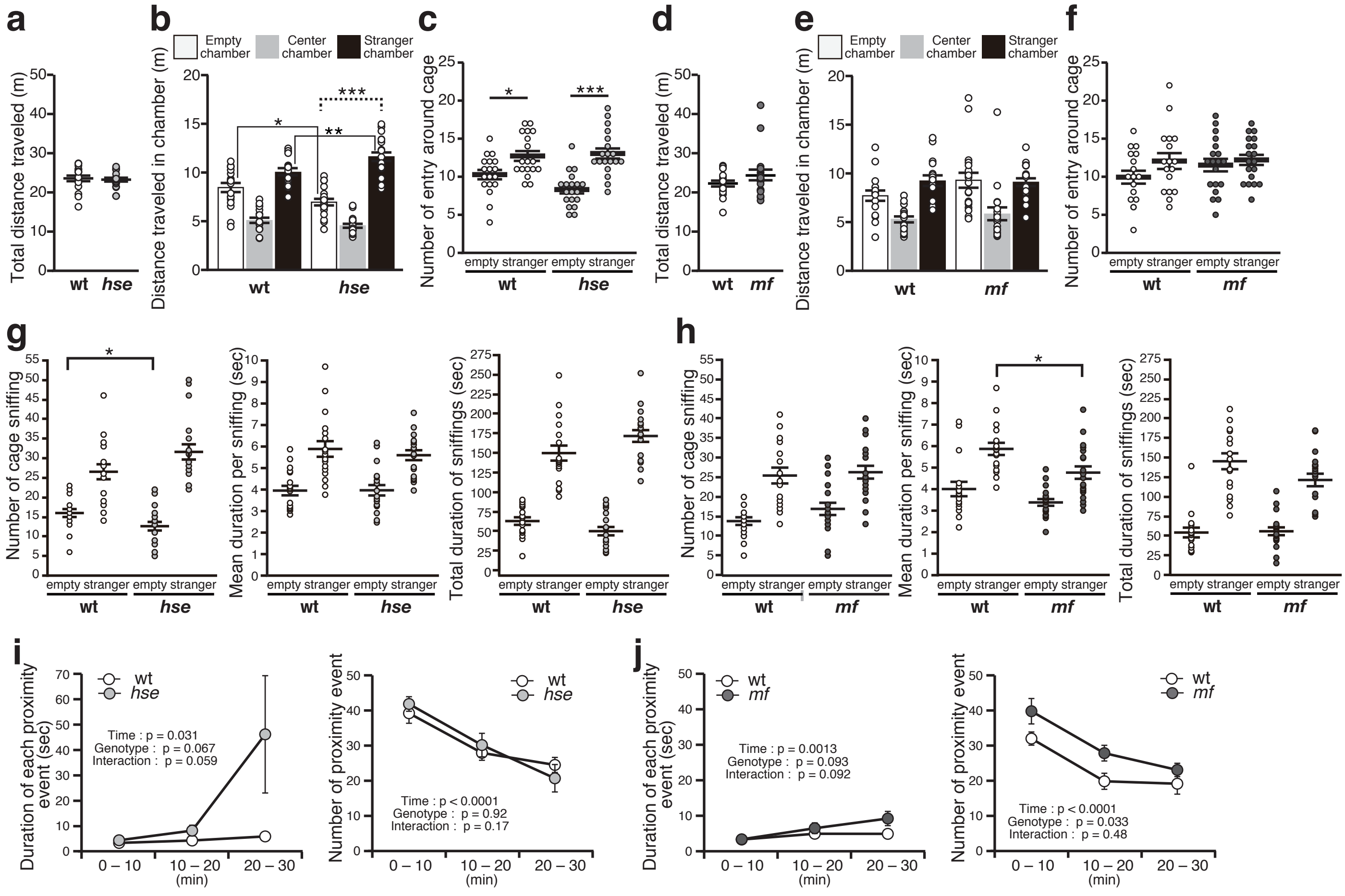

Supplementary Fig. 8: Social behavior of *Nlgn3*^{hse} and *Nlgn3*^{mf} mice. **a–h**, Total distance (**a** and **d**), distance traveled in each chamber (**b** and **e**), number of entry around empty and stranger cages (**c** and **f**), and cage sniffing behavior of *Nlgn3*^{hse} and their littermate wild-type mice (**a–c, g**) (N = 19 and 18 for *Nlgn3*^{hse} and littermate wild-type, respectively) and *Nlgn3*^{mf} and their littermate wild-type mice (**d–f**, **h**) (N = 19 and 17 for *Nlgn3*^{mf} and littermate wild-type, respectively) during three-chamber sociability tests. For cage sniffing behavior (**g** and **h**), number of cage sniffing (left), mean duration of each sniffing (middle), and total duration of sniffing (right) are quantified. **i** and **j**, Averaged duration of each proximity event (left) and number of proximity events (right) during 30 minutes reciprocal social interaction test of *Nlgn3*^{hse} mutant mice (**i**) (N = 13 and 14 pairs for *Nlgn3*^{hse} and littermate wild-type, respectively) and *Nlgn3*^{mf} mutant mice (**j**) (N = 14 and 13 pairs for *Nlgn3*^{mf} and littermate wild-type, respectively) are presented. Data are mean ± s.e.m. *$p < 0.05$, **$p < 0.01$ and ***$p < 0.001$, Two-sided Student' s t-test in **b, c, g** and **h** and Bonferroni post-test in **b** (comparison with dotted line). See Supplementary Table 4 for additional statistics and exact p values.

Supplementary Table 1. A list of membrane proteins identified from PTPδA3B⁻-Fc coated beads.

Bait	Protein[a]	Probability[b]	Score[c]	Hits[d]
PTPδ-A3B⁻-Fc	GluN2B	5.37E-09	44.2	7
	NLGN3	7.66E-09	10.22	2
	TMEM14A	3.68E-06	10.08	1
	NCAM1	2.91E-05	90.22	10

Protein[a], proteins with probability value (<1.0E-04) are listed; Probability[b], probability (protein) of finding a match as good as or better than the observed match by chance; Score[c], SEQUEST scores; Hits[d], number of unique parent peptides found.

Supplementary Table 2. Data collection and refinement statistics.

	NLGN3 ECD	NLGN3 ECD–PTPδ Ig1–Fn1
Data collection		
Wavelength (Å)	1.0000	1.0000
Resolution (Å)	50.0–2.76 (2.81–2.76)	50.0–3.85 (3.92–3.85)
Space group	$P2_12_12_1$	$P3_212$
Cell dimensions		
a, *b*, *c* (Å)	65.8, 167.1, 177.9	96.1, 96.1, 371.6
α, *β*, *γ* (°)	90.0, 90.0, 90.0	90.0, 90.0, 120.0
Completeness (%)	99.1 (98.7)	100 (100)
$CC_{1/2}$	(0.518)	(0.570)
R_{sym} (%)	11.1 (39.8)	19.6 (182.1)
$I/\sigma I$	12.2 (1.8)	15.1 (0.98)
Redundancy	7.7 (4.8)	18.7 (14.3)
Refinement		
Resolution (Å)	49.7–2.76	49.7–3.85
No. reflections	50,376	19,070
No. atoms		
Protein	8,374	7,217
Sugar	56	56
Water	59	–
R_{work}/R_{free}	0.218 / 0.251	0.253 / 0.289
R.m.s.d.		
Bond lengths (Å)	0.003	0.002
Bond angles (°)	0.871	0.499
Average *B*-factor ($Å^2$)		
Protein	70.5	189.2
Sugar	128.2	239.0
Water	59.4	–
Ramachandran plot		
Most favored (%)	97.1	94.8
Disallowed (%)	0	0

Values in parentheses are for the highest-resolution shell.

Supplementary Table 3. Primer and ssODN sequences used in this study.

name	sequence
**Construction of expression vectors**	
Nlgn2-Cla-S	5'-ATCGATAGGCAGCCTCGGGGAGGAG-3'
Nlgn2-Sal-A	5'-GTCGACCTATACCCGAGTGGTGGAGTG-3'
Nlgn3-ERI-S2	5'-GAATTCAACCCAGGCCCCGGCACCC-3'
Nlgn3-Sal-A	5'-GTCGACCTATACACGGGTAGTGGAGTGTG-3'
Nlgn4-ERI-S	5'-GATATCGCGCTCGCCACGCTATACC-3'
Nlgn4-RV-A	5'-GAATTCATCCCCGCCGCCGCCGCC-3'
**Crystallization**	
Xho-Nlgn3-S	5'-CCGGCTCGAGGCCACCATGTGGCTGCAGCCCTCGCT-3'
Not-Nlgn3-2052	5'-CCGGATGCGGCCGCTCAGTGATGGTGATGGTGATGTTCAGTGGAGTAGTCACGAG-3'
**Generation of neuron-specific NLGN3 KO mice**	
Nlgn3-GT-A	5’-TGTACCAGGAATGGGAAGCAG-3’
Nlgn3-GT-B	5’-GGTCAGAGCTGTCATTGTCAC-3’
Nlgn3-GT-C	5’-AGCAAGAGCTCTACTACCTCC-3’
Cre-S	5’-AGGTTCGTTCACTCATGGA-3’
Cre-A	5’-TCGACCAGTTTAGTTACCC-3’
**Generation of Nlgn3mf and Nlgn3hse knock-in mice**	
gRNA-E6T2	5’-CAGAATGATGAGACAGTGTAAGG-3’
E6-ssODN1	5'-ACTGTCTTTGGCTCTGGCATCGGTGCATCCTGTGTCAGTCTCCTTACTCTCTCTCATGC AGCTGCGGGTGAGTAACTCTTGAGCATCAACAGAGAATAGCTTTTTCTTTC-3’
gRNA-E8T2	5’-TATAGTGGAACATGTCATGCAGG-3’
E8-ssODN	5'-AAAGGTAGCCTTTTGGAAACACCTGGTGCCCCACCTGTACAACCTTCATGACGCGGCC CACTACACATCCACGACCACCAAAGTGCCGCCCCCGGACACCACCCACAGCT-3’
Nlgn3-E6-U2	5’-CTAGGTTTCCTGAGCACTGGAG-3’
Nlgn3-E6-L2	5’-ATAGAGGGAGTTCCAGGACAGC-3’
Nlgn3-E8-U2	5’-GGCTGAAACCAAGGGTTCGTG-3’,
Nlgn3-E8-L2	5’-TGCTGTGAGGCGAAGTGTGT-3’
**Real-time PCR**	
Ptprd-725-S	5’-CACCAAGATTCTCTATCCCAC-3’
Ptprd-941-A	5’-GTCGACATAGCAACACAGGTG-3’
Gapdh-S	5’-ACATCATCCCTGCATCCACTGG-3’
Gapdh-A	5’-TCCTCAGTGTAGCCCAAGATGC-3’
Ptprd-460-S	5’-TGTGCAGCCAGCGGTAATCCG-3’
Ptprd-1907-A	5’-ATCTCCCAAGACAGCAGCACTG-3’
PtprdA3-S	5’-TTACGATCAGAATCTATTGGAGCC-3’
PtprdB-A	5’-GGGACACGGCGAACTCTGAC-3’

Supplementary Table 4. Exact p values for the indicated statistical tests.

Fig. 1b
Staining signal intensity
Tukey-Kramer HSD test (two-sided)

	p-Value (Prob>|t|)
Shank2, PTPδ A9B+ vs Fc	<.0001
Shank2, PTPδ A6B+ vs Fc	0.0102
Shank2, PTPδ A3B+ vs Fc	0.0402
Shank2, PTPδ A-B+ vs Fc	0.384
Shank2, PTPδ A9B- vs Fc	<.0001
Shank2, PTPδ A6B- vs Fc	<.0001
Shank2, PTPδ A3B- vs Fc	<.0001
Shank2, PTPδ A-B- vs Fc	0.484
Shank2, NRXN1β vs Fc	<.0001
Gephyrin, PTPδ A9B+ vs Fc	1
Gephyrin, PTPδ A6B+ vs Fc	0.998
Gephyrin, PTPδ A3B+ vs Fc	1
Gephyrin, PTPδ A-B+ vs Fc	0.994
Gephyrin, PTPδ A9B- vs Fc	<.0001
Gephyrin, PTPδ A6B- vs Fc	<.0001
Gephyrin, PTPδ A3B- vs Fc	0.0001
Gephyrin, PTPδ A-B- vs Fc	0.132
Gephyrin, NRXN1β vs Fc	<.0001

Fig. 2c
Binding activity (Fc signal/FLAG signal)
Dunnett test (two-sided)
vs. Control group (Fc)

	p-Value (Prob>|t|)
PTPδ A9B+	1
PTPδ A3B+	1
PTPδ A6B+	1
PTPδ A–B+	1
PTPδ A9B–	0.0013
PTPδ A6B–	<.0001
PTPδ A3B–	<.0001
PTPδ A–B–	0.97
NRXN1β	<.0001

Tukey-Kramer HSD test (two-sided)
vs. PTPδ A3B+

	p-Value (Prob>|t|)
PTPδ A3B+ vs PTPδ A9B+	<.0001
PTPδ A3B+ vs PTPδ A6B+	<.0001
PTPδ A3B+ vs PTPδ A3B+	<.0001
PTPδ A3B+ vs PTPδ A–B+	<.0001
PTPδ A3B+ vs PTPδ A9B–	<.0001
PTPδ A3B+ vs PTPδ A6B–	<.0001
PTPδ A3B+ vs PTPδ A–B–	<.0001

Fig. 2g
Signal intensity
Tukey-Kramer HSD test (two-sided)

	p-Value (Prob>|t|)
Shank2, Fc (wt) vs Fc (KO)	1
Shank2, PTPδ A9B+ (wt) vs PTPδ A9B+ (KO)	0.999
Shank2, PTPδ A3B– (wt) vs PTPδ A3B– (KO)	<.0001
Shank2, PTPδ A9B+ (wt) vs Fc (wt)	<.0001
Shank2, PTPδ A9B+ (KO) vs Fc (wt)	<.0001
Shank2, PTPδ A3B– (wt) vs Fc (wt)	<.0001

Shank2, PTPδ A3B– (KO) vs Fc (wt)	0.915
Shank2, PTPδ A9B+ (wt) vs Fc (KO)	<.0001
Shank2, PTPδ A9B+ (KO) vs Fc (KO)	<.0001
Shank2, PTPδ A3B– (wt) vs Fc (KO)	<.0001
Shank2, PTPδ A3B– (KO) vs Fc (KO)	0.989
Shank2, PTPδ A9B+ (wt) vs PTPδ A3B– (wt)	1
Shank2, PTPδ A9B+ (wt) vs PTPδ A3B– (KO)	<.0001
Shank2, PTPδ A9B+ (KO) vs PTPδ A3B– (KO)	<.0001
Shank2, PTPδ A3B– (wt) vs PTPδ A9B+ (KO)	1
Gephyrin, Fc (wt) vs Fc (KO)	1
Gephyrin, PTPδ A9B+ (wt) vs PTPδ A9B+ (KO)	0.999
Gephyrin, PTPδ A3B– (wt) vs PTPδ A3B– (KO)	<.0001
Gephyrin, PTPδ A9B+ (wt) vs Fc (wt)	0.997
Gephyrin, PTPδ A9B+ (KO) vs Fc (wt)	1
Gephyrin, PTPδ A3B– (wt) vs Fc (wt)	<.0001
Gephyrin, PTPδ A3B– (KO) vs Fc (wt)	0.998
Gephyrin, PTPδ A3B– (wt) vs Fc (KO)	<.0001
Gephyrin, PTPδ A9B+ (wt) vs Fc (KO)	0.998
Gephyrin, PTPδ A9B+ (KO) vs Fc (KO)	1
Gephyrin, PTPδ A3B– (KO) vs Fc (KO)	0.998
Gephyrin, PTPδ A3B– (wt) vs PTPδ A9B+ (wt)	<.0001
Gephyrin, PTPδ A9B+ (wt) vs PTPδ A3B– (KO)	1
Gephyrin, PTPδ A3B– (wt) vs PTPδ A9B+ (KO)	<.0001
Gephyrin, PTPδ A3B– (wt) vs PTPδ A9B+ (KO)	1

Fig. 2h

Cell surface PTPδ A3B- Fc signal intensity

Analysis of Variance two-sided

Source	DF	F ratio	p-value (Prob>F)
Model	20	16.4378	<.0001
Error	273		
C.Total	293		

Effect tests

Source	DF	F Ratio	p-value (Prob>F)
Competitor Concentration	6	21.9313	<.0001
Competitors	2	62.8005	<.0001
Competitor Concentration*Competitors	12	5.9639	<.0001

Tukey-Kramer HSD test (two-sided)

	p-Value (Prob>|t|)
0 μM PTPδ A3B– vs 0.1 uM 0 μM PTPδ A3B–	0.0002
0 μM PTPδ A3B– vs 0.3 μM PTPδ A3B–	<.0001
0 μM PTPδ A3B– vs 1 μM PTPδ A3B–	<.0001
0 μM PTPδ A3B– vs 3 μM PTPδ A3B–	<.0001
0 μM NRXN1β vs 1 uM NRXN1β	<.0001
0 μM NRXN1β vs 3 uM NRXN1β	<.0001

Fig. 4g

NRXN1β-Fc pulldown

Tukey-Kramer HSD test two-sided

	p-Value (Prob>|t|)
WT vs HSE/AAA	0.0005
WT vs DEN/AAA	0.0003

PTPδ A3B–-Fc pulldown

Tukey-Kramer HSD test two-sided

	p-Value (Prob>|t|)
WT vs MF/IS	<.0001
WT vs MF/AA	<.0001
WT vs DEN/AAA	<.0001

Fig. 5a

Signal intensity

NRXN1β beads

t-test two-sided

	p-Value (Prob>|t|)
Shank2, wt vs Nlgn3hse	0.00028
Gephyrin, wt vs Nlgn3hse	0.0128

PTPδ A3B– beads

t-test two-sided

	p-Value (Prob>|t|)
Shank2, wt vs Nlgn3hse	0.584
Gephyrin, wt vs Nlgn3hse	0.0173

Fig. 5b

Signal intensity

NRXN1β beads

t-test two-sided

	p-Value (Prob>|t|)
Shank2, wt vs Nlgn3mf	0.0979
Gephyrin, wt vs Nlgn3mf	0.0007969

PTPδ A3B– beads

t-test two-sided

	p-Value (Prob>|t|)
Shank2, wt vs Nlgn3mf	1.50E-08
Gephyrin, wt vs Nlgn3mf	4.94E-09

Fig. 5c

NLGN3 signal intensity

Analysis of Variance

Source	DF	F ratio	p-value (Prob>F)
Bead Genotype	5	34.5694	<.0001
Error	194		
C.Total	199		

Tukey-Kramer HSD test (two-sided)

	p-Value (Prob>|t|)
NRXN1β beads, wt vs Nlgn3hse	0.0221
PTPδ A3B– beads, wt vs Nlgn3hse	0.0012

Fig. 5d

NLGN3 signal intensity

Analysis of Variance

Source	DF	F ratio	p-value (Prob>F)
Bead Genotype	5	19.3546	<.0001
Error	156		
C.Total	161		

Tukey-Kramer HSD test (two-sided)

	p-Value (Prob>|t|)
NRXN1β beads, wt vs Nlgn3mf	<.0001
PTPδ A3B– beads, wt vs Nlgn3mf	0.0008

Fig. 5e

co-IP Efficiency (PTPδ/NLGN3)

t-test (two-sided)

	p-Value (Prob>|t|)
wt vs Nlgn3hse	0.0062
wt vs Nlgn3mf	0.00026

Fig. 5f
Time spent in chamber (sec)
Bonferroni test (two-sided)

	p-value
wt, Empty vs Center	2.64E-09
wt, Empty vs Stranger	0.0019
wt, Center vs Empty	7.71E-13
hse, Empty vs Center	4.14E-09
hse, Empty vs Stranger	1.34E-06
hse, Center vs Empty	7.73E-14

t-test (two-sided)

	p-Value (Prob>|t|)
Empty chamber, wt vs hse	0.0326
Center chamber, wt vs hse	0.175
Stranger chamber, wt vs hse	0.0138

Fig. 5g
Preference index
Wilcoxon (Mann U Tests (Rank Sums))

	p-value (Prob>|Z|)
wt vs hse	0.0102

Fig. 5h
Time spent in chamber (sec)
Bonferroni test (two-sided)

	p-value
wt, Empty vs Center	2.33E-07
wt, Empty vs Stranger	0.0071
wt, Center vs Empty	7.55E-10
mf, Empty vs Center	2.61E-07
mf, Empty vs Stranger	0.111
mf, Center vs Empty	1.51E-10

t-test (two-sided)

	p-Value (Prob>|t|)
Empty chamber, wt vs mf	0.292
Center chamber, wt vs mf	0.743
Stranger chamber, wt vs mf	0.225

Fig. 5i
Preference index
Wilcoxon (Mann U Tests (Rank Sums))

	p-value (Prob>|Z|)
wt vs mf	0.506

Fig. 5j
Total duration of proximity event (sec)
Anslysis of Variance：Total duration of proximity event (sec)

	DF	F ratio	p-value (Prob>F)
Genotype	1	9.133	0.0057
Subject（Group）	25		
Total duration	2	4.363	0.0179
Total duration * Genotype	2	3.816	0.0287
Total duration * Subject（Group）	50		

t-test (two-sided)

	p-Value (Prob>|t|)

0-10 min, wt vs hse	0.0448
10-20 min, wt vs hse	0.0148
20-30 min, wt vs hse	0.0165

Fig. 5l

Total duration of proximity event (sec)

Anslysis of Variance：Total duration of proximity event (sec)

	DF	F ratio	p-value (Prob>F)
Genotype	1	8.396	0.0077
Subject（Group）	25		
Totalduration	2	2.035	0.1414
Totalduration * Genotype	2	4.075	0.0229
Totalduration * Subject（Group）	50		

t-test (two-sided)

	p-Value (Prob>|t|)
0-10 min, wt vs mf	0.111
10-20 min, wt vs mf	0.0114
20-30 min, wt vs mf	0.013

Fig. 5m

Elevated head-head contact

Anslysis of Variance：Elevated head-head contact two-sided

	DF	F ratio	p-value (Prob>F)
Genotype	1	2.355	0.1374
Subject（Group）	25		
Head to head	2	0.49	0.6155
Head to head * Genotype	2	5.281	0.0083
Head to head * Subject（Group）	50		

t-test (two-sided)

	p-Value (Prob>|t|)
0-10 min, wt vs mf	0.796
10-20 min, wt vs mf	0.0072
20-30 min, wt vs mf	0.533

Fig. 5p

Rotarod, Terminal speed (rpm)

Anslysis of Variance：Trial 1-12

Group	DF	F ratio	p-value (Prob>F)
Genotpe	1	12.503	0.0007
Subject（Group）	70		
Trial1-12	11	29.091	<.0001
Trial1-12 * Genotpe	11	1.274	0.2349
Trial1-12 * Subject(Group）	770		

t-test (two-sided)

	p-Value (Prob>|t|)
Trial 1, wt vs mf	0.207
Trial 2, wt vs mf	0.00099
Trial 3, wt vs mf	0.04469
Trial 4, wt vs mf	0.00489
Trial 5, wt vs mf	0.00229
Trial 6, wt vs mf	0.0263
Trial 7, wt vs mf	0.001884
Trial 8, wt vs mf	0.00153
Trial 9, wt vs mf	0.00287
Trial 10, wt vs mf	0.0108

Trial 11, wt vs mf	0.0294
Trial 12, wt vs mf	0.00625

Fig. 6b
Pulldown efficiency (% WT)
Tukey-Kramer HSD test (two-sided)

	p-Value (Prob>|t|)
NRXN1β-Fc, WT vs R/C	0.0246
NRXN1β-Fc, WT vs DEN	0.0026
PTPδ A3B– -Fc, WT vs R/C	0.0008
PTPδ A3B– -Fc, WT vs DEN	0.0002

Fig. 6c
Shank2 and Gephyrin signal intensity
Tukey-Kramer HSD test (two-sided)

	p-Value (Prob>|t|)
Shank2, Fc, wt vs R/C	1
Shank2, NRXN1β, wt vs R/C	0.0832
Shank2, PTPδ A3B–, wt vs R/C	0.0231
Gephyrin , Fc, wt vs R/C	1
Gephyrin , NRXN1β, wt vs R/C	<.0001
Gephyrin , PTPδ A3B–, wt vs R/C	0.0177

Fig. 6d
Protein levels
t-test (two-sided)

	p-Value (Prob>|t|)
PSD95, wt vs hse	0.85
VGluT1, wt vs hse	0.787
Gephyrin, wt vs hse	0.776
VGAT, wt vs hse	0.562
VAMP2, wt vs hse	0.854
PSD95, wt vs mf	0.99
VGluT1, wt vs mf	0.57
Gephyrin, wt vs mf	0.000468
VGAT, wt vs mf	0.00222
VAMP2, wt vs mf	0.47

Fig. 6e
Frequency and amplitude of mEPSC and mIPSC
t-test (two-sided)

mEPSC frequency, wt vs hse	0.555
mEPSC amplitude, wt vs hse	0.1645
mIPSC frequency, wt vs hse	0.399
mIPSC amplitude, wt vs hse	0.0557

Fig. 6f
Frequency and amplitude of mEPSC and mIPSC
t-test (two-sided)

mEPSC frequency, wt vs mf	0.0426
mEPSC amplitude, wt vs mf	0.941
mIPSC frequency, wt vs mf	0.00645
mIPSC amplitude, wt vs mf	0.218351851

Supplementary Fig. 1b
Shank2 signal intensity
Tukey-Kramer HSD test (two-sided)

	p-Value (Prob>|t|)
WT (PTPδ A9B+) vs WT (Fc)	<.0001
WT (PTPδ A9B+) vs DKO (PTPδ A9B+)	<.0001

DKO (PTPδ A9B+) vs WT (Fc)	0.0011

Supplementary Fig. 1d
Shank2 and Gephyrin signal intensity
Tukey-Kramer HSD test (two-sided)

	p-Value (Prob>|t|)
Shank2, WT (PTPδ A3B–) vs DKO (PTPδ A3B–)	0.545
Shank2, WT (PTPδ A3B–) vs WT (Fc)	0.0023
Shank2, DKO (PTPδ A3B–) vs WT (Fc)	<.0001
Gephyrin, WT (PTPδ A3B–) vs DKO (PTPδ A3B–)	0.922
Gephyrin, WT (PTPδ A3B–) vs WT (Fc)	0.0022
GephyrinDKO (PTPδ A3B–) vs WT (Fc)	0.0066

Supplementary Fig. 2a
Binding activity (Fc signal/FLAG-NLGN3 singnal)
Control group = Fc
Dunnett test (two-sided)

	p-Value (Prob>|t|)
PTPδ A3B– Fc	<.0001
LAR A6B+ Fc	1
LAR A6B– Fc	0.0859
LAR A–B+ Fc	1
LAR A–B– Fc	1
PTPσ A–B+ Fc	1
PTPσ A–B– Fc	0.964
PTPσ A9B+ Fc	1

Supplementary Fig. 2b
Binding activity
Control group = Fc
Dunnett test (two-sided)

	p-Value (Prob>|t|)
NRXN1β vs NLGN1(–)	<.0001
NRXN1β vs NLGN1B	0.0724
NRXN1β vs NLGN1A	0.0011
NRXN1β vs NLGN1AB	0.456
NRXN1β vs NLGN2A	<.0001
NRXN1β vs NLGN3 (–)	<.0001
NRXN1β vs NLGN3A	<.0001
NRXN1β vs NLGN4 (–)	0.944
PTPδ A3B– vs NLGN1 (–)	0.999
PTPδ A3B– vs NLGN1B	0.671
PTPδ A3B– vs NLGN1A	0.392
PTPδ A3B– vs NLGN1AB	0.365
PTPδ A3B– vs NLGN2A	0.996
PTPδ A3B– vs NLGN3 (–)	<.0001
PTPδ A3B– vs NLGN3A	<.0001
PTPδ A3B– vs NLGN4 (–)	0.369

Supplementary Fig. 2e
Binding activity (Fc siganl/FLAG signal)
NRXN1β vs NLGN3-mutants
Tukey-Kramer HSD test (two-sided)

	p-Value (Prob>|t|)
NLGN3-Fc vs Fc	<.0001
NLGN3-Fc vs NLGN3-LND-Fc	<.0001
NLGN3-LND-Fc vs Fc	0.453

PTPδ A3B– vs NLGN3-mutants
Tukey-Kramer HSD test (two-sided)

	p-Value (Prob>|t|)
NLGN3-Fc vs Fc	<.0001
NLGN3-Fc vs NLGN3-LND-Fc	<.0001
NLGN3-LND-Fc vs Fc	<.0001

Supplementary Fig. 2f
Binding activity (Cell surface PTPδ A3B– Fc singnals)
Control group = 0 μM MDGA1-myc-His
Dunnett test (two-sided)

	p-Value (Prob>|t|)
0.01 μM MDGA1-myc-His	1
0.03 μM MDGA1-myc-His	0.998
0.1 μM MDGA1-myc-His	0.952
0.3 μM MDGA1-myc-His	0.218
1 μM MDGA1-myc-His	0.0317
3 μM MDGA1-myc-His	0.0065

Supplementary Fig. 3e
Shank2 signal intensity
t-test (two-sided)

	p-Value (Prob>|t|)
PTPδ A9B+, wt vs KO	0.594
PTPδ A6B+, wt vs KO	0.325
PTPδ A3B+, wt vs KO	0.854
PTPδ A–B+, wt vs KO	0.637
PTPδ A9B–, wt vs KO	1.59E-06
PTPδ A6B–, wt vs KO	7.24E-07
PTPδ A3B–, wt vs KO	0.0000679
PTPδ A–B–, wt vs KO	0.000157
Fc, wt vs KO	0.934

Supplementary Fig. 3f
Gephyrin signal intensity
t-test (two-sided)

	p-Value (Prob>|t|)
PTPδ A9B+, wt vs KO	0.308
PTPδ A6B+, wt vs KO	0.742
PTPδ A3B+, wt vs KO	0.931
PTPδ A–B+, wt vs KO	0.692
PTPδ A9B–, wt vs KO	0.000356
PTPδ A6B–, wt vs KO	0.000611
PTPδ A3B–, wt vs KO	0.0101
PTPδ A–B–, wt vs KO	0.0000405
Fc, wt vs KO	0.551

Supplementary Fig. 6d
Binding activity (Fc signal/ FLAG signal)
Tukey-Kramer HSD test (two-sided)

	p-Value (Prob>|t|)
FLAG-NRXN1β, WT-Fc vs Fc	0.0001
FLAG-NRXN1β, WT-Fc vs HSE-Fc	0.0003
FLAG-NRXN1β, HSE-Fc vs Fc	0.973
FLAG-NRXN2β, WT-Fc vs Fc	<.0001
FLAG-NRXN2β, WT-Fc vs HSE-Fc	<.0001
FLAG-NRXN2β, HSE-Fc vs Fc	0.999
FLAG-NRXN3β, WT-Fc vs Fc	<.0001
FLAG-NRXN3β, WT-Fc vs HSE-Fc	<.0001
FLAG-NRXN3β, HSE-Fc vs Fc	0.983

Supplementary Fig. 6e
Binding activity (Fc signal/ FLAG signal)

Tukey-Kramer HSD test (two-sided)

<table>
<tr><th></th><th>p-Value (Prob>|t|)</th></tr>
<tr><td>0.15 μM, WT vs HSE/AAA</td><td>0.977</td></tr>
<tr><td>0.15 μM, WT vs MF/IS</td><td><.0001</td></tr>
<tr><td>0.15 μM, WT vs MF/AA</td><td><.0001</td></tr>
<tr><td>0.15 μM, WT vs DEN/AAA</td><td><.0001</td></tr>
<tr><td>0.15 μM, WT vs Fc</td><td><.0001</td></tr>
<tr><td>0.15 μM, MF/IS vs MF/AA</td><td>0.0087</td></tr>
<tr><td>0.15 μM, MF/IS vs DEN/AAA</td><td><.0001</td></tr>
<tr><td>0.15 μM, MF/IS vs Fc</td><td><.0001</td></tr>
<tr><td>0.15 μM, MF/AA vs DEN/AAA</td><td>0.0235</td></tr>
<tr><td>0.15 μM, MF/AA vs Fc</td><td>0.0314</td></tr>
</table>

Supplementary Fig. 7d and 7g

NLGN3 protein levels

t-test (two-sided)

<table>
<tr><th></th><th>p-Value (Prob>|t|)</th></tr>
<tr><td>wt vs hse</td><td>0.465</td></tr>
<tr><td>wt vs mf</td><td>0.926</td></tr>
</table>

Supplementary Fig. 7h and 7i

Staining signal intensity

t-test (two-sided)

<table>
<tr><th></th><th>p-Value (Prob>|t|)</th></tr>
<tr><td>Shank2 signal, wt vs hse</td><td>0.315</td></tr>
<tr><td>Gephyrin signal, wt vs hse</td><td>0.171</td></tr>
<tr><td>Gephyrin/Shank2 signal ratio, wt vs hse</td><td>0.719</td></tr>
<tr><td>Shank2 signal, wt vs mf</td><td>0.133</td></tr>
<tr><td>Gephyrin signal, wt vs mf</td><td>0.596</td></tr>
<tr><td>Gephyrin/Shank2 signal ratio, wt vs mf</td><td>0.137</td></tr>
</table>

Supplementary Fig. 7j and 7k

VGAT/VGlut1 Ratio

Wilcoxon (Mann-U (Rank Sums))

<table>
<tr><th></th><th>p-value (Prob>|Z|)</th></tr>
<tr><td>Somatosensory Cortex, wt vs hse</td><td>0.0606</td></tr>
<tr><td>medial Prefrontal Cortex, wt vs hse</td><td>0.286</td></tr>
<tr><td>Somatosensory Cortex, wt vs mf</td><td>0.0074</td></tr>
<tr><td>medial Prefrontal Cortex, wt vs mf</td><td>0.0009</td></tr>
</table>

Supplementary Fig. 8a and 8d

Total distance traveled (m)

t-test (two-sided)

<table>
<tr><th></th><th>p-Value (Prob>|t|)</th></tr>
<tr><td>wt vs hse</td><td>0.607</td></tr>
<tr><td>wt vs mf</td><td>0.206</td></tr>
</table>

Supplementary Fig. 8b

Distance traveled in chamber (m)

Bonferroni test (two-sided)

	p-value
wt, Empty vs Center	3.94E-06
wt, Empty vs Stranger	0.0671
wt, Center vs Empty	2.33E-09
hse, Empty vs Center	1.91E-05
hse, Empty vs Stranger	9.06E-06
hse, Center vs Empty	8.06E-12

t-test (two-sided)

	p-value
Empty chamber	0.0132

Center chamber	0.113
Stranger chamber	0.00919

Supplementary Fig. 8c
Number of entry around cage
t-test (two-sided)

	p-value
wt, Empty vs Stranger	0.0172
hse, Empty vs Stranger	0.000145

Supplementary Fig. 8e
Distance traveled in chamber (m)
Bonferroni test (two-sided)

	p-value
wt, Empty vs Center	0.0015
wt, Empty vs Stranger	0.338
wt, Center vs Empty	1.18E-05
mf, Empty vs Center	1.29E-04
mf, Empty vs Stranger	1
mf, Center vs Empty	9.48E-04

t-test (two-sided)

	p-value
Empty chamber	0.105
Center chamber	0.463
Stranger chamber	0.844

Supplementary Fig. 8f
Number of entry around cage
t-test (two-sided)

	p-value
wt, Empty vs Stranger	0.18
mf, Empty vs Stranger	0.495

Supplementary Fig. 8g
Cage sniffing during 3-chamber social test, wt vs hse
Student's t-test (two-sided)

	p-value (Prob>|t|)
Number of empty cage sniffing	0.0314
Number of stranger cage sniffing	0.0717
Mean duration of empty cage sniffing	0.965
Mean duration of stranger cage sniffing	0.5
Total duration of empty sniffing	0.0836
Total duration of stranger sniffing	0.0772

Supplementary Fig. 8h
Cage sniffing during 3-chamber social test, wt vs mf
Student's t-test (two-sided)

	p-value (Prob>|t|)
Number of empty cage sniffing	0.101
Number of stranger cage sniffing	0.743
Mean duration of empty cage sniffing	0.106
Mean duration of stranger cage sniffing	0.0104
Total duration of empty sniffing	0.84
Total duration of stranger sniffing	0.0745
