# Supplementary material for: Canonical versus non-canonical transsynaptic signaling of neuroligin 3 tunes development of sociality in mice
Source: Nat Commun. 2021 Mar 23;12:1848. doi: 10.1038/s41467-021-22059-6 (PMC7988105; doi:10.1038/s41467-021-22059-6)
Supplement: Supplementary file 3 — Reporting Summary [file 41467_2021_22059_MOESM3_ESM.pdf]

nature research

Corresponding author(s): Tomoyuki Yoshida & Shuya Fukai

Last updated by author(s): Feb 9, 2021

Reporting Summary

Nature Research wishes to improve the reproducibility of the work that we publish. This form provides structure for consistency and transparency in reporting. For further information on Nature Research policies, see our Editorial Policies and the Editorial Policy Checklist.

Statistics

For all statistical analyses, confirm that the following items are present in the figure legend, table legend, main text, or Methods section.

n/a	Confirmed	
☐	☒	The exact sample size (*n*) for each experimental group/condition, given as a discrete number and unit of measurement
☐	☒	A statement on whether measurements were taken from distinct samples or whether the same sample was measured repeatedly
☐	☒	The statistical test(s) used AND whether they are one- or two-sided<br>*Only common tests should be described solely by name; describe more complex techniques in the Methods section.*
☒	☐	A description of all covariates tested
☐	☒	A description of any assumptions or corrections, such as tests of normality and adjustment for multiple comparisons
☐	☒	A full description of the statistical parameters including central tendency (e.g. means) or other basic estimates (e.g. regression coefficient) AND variation (e.g. standard deviation) or associated estimates of uncertainty (e.g. confidence intervals)
☐	☒	For null hypothesis testing, the test statistic (e.g. F, t, r) with confidence intervals, effect sizes, degrees of freedom and P value noted<br>*Give P values as exact values whenever suitable.*
☒	☐	For Bayesian analysis, information on the choice of priors and Markov chain Monte Carlo settings
☒	☐	For hierarchical and complex designs, identification of the appropriate level for tests and full reporting of outcomes
☒	☐	Estimates of effect sizes (e.g. Cohen's *d*, Pearson's *r*), indicating how they were calculated

Our web collection on statistics for biologists contains articles on many of the points above.

Software and code

Policy information about availability of computer code

Data collection	Crystallographic data collection: BSS (SPring-8 BL41XU)<br>Electrophysiological data collection & processing: pCLAMP 8 (Axon Instruments)<br>Behavioral data collection: Image J 1.47a (NIH)
Data analysis	Crystallographic data processing: CCP4 program suite, HKL2000, Molrep (Acta. Crystallogr. D Biol Crystallogr. 66, 22–25 (2010)), Coot (Acta. Crystallogr. D Biol. Crystallogr. 60, 2126–2132 (2004)), PISA (J. Mol. Biol. 372, 774–797 (2007)), MolProbity (Nucleic Acids Res. 35, W375–383 (2007)), and PyMOL (Schrödinger, LLC)<br>ITC analysis: Origin (MicroCal)<br>SEC-MALS analysis: ASTRA (Wyatt Technology)<br>Statistical analysis: JMP Pro (v15.0.0, SAS inc.)<br>Densitometric analysis: Image J 1.46 (NIH)

For manuscripts utilizing custom algorithms or software that are central to the research but not yet described in published literature, software must be made available to editors and reviewers. We strongly encourage code deposition in a community repository (e.g. GitHub). See the Nature Research guidelines for submitting code & software for further information.

Data

Policy information about availability of data

All manuscripts must include a data availability statement. This statement should provide the following information, where applicable:

- Accession codes, unique identifiers, or web links for publicly available datasets
- A list of figures that have associated raw data
- A description of any restrictions on data availability

The coordinates and structure factors of apo-NLGN3 ECD and the NLGN3 ECD–PTPδ Ig1–Fn1 complex have been deposited in the Protein Data Bank under the accession codes 7CEE and 7CEG, respectively. The source data and detailed statistics underlying Fig. 1b, 2c, 2g, 2h, 4g, 5a–m, 5o, 5p, 6b–f, Supplementary Fig. 1b, 1d, 2a–f, 3e, 3f, 6d, 6e, 7d, 7g–k, and 8a–j, are provided as a Source Data file. Other data, mouse strains and custom-made rabbit anti-VGluT1 antibody generated in this study are available from the corresponding authors upon reasonable request.

Field-specific reporting

Please select the one below that is the best fit for your research. If you are not sure, read the appropriate sections before making your selection.

[x] Life sciences [ ] Behavioural & social sciences [ ] Ecological, evolutionary & environmental sciences

For a reference copy of the document with all sections, see nature.com/documents/nr-reporting-summary-flat.pdf

Life sciences study design

All studies must disclose on these points even when the disclosure is negative.

Sample size	For behavioral analysis, the sample size was set to approximately 20 for each group, which was determined to detect a difference of 1.0 effect size (Cohen's d) with at least 80% power at the 0.05 level of significance (two-side test).<br>For biochemical and cell-based analyses, no statistical methods were used to predetermine sample size. However, suitable sample sizes were estimated based on previously reported studies with similar experimental designs (e.g. Nat Neurosci. 15,389-398 (2012); Cell 173,735-748 (2018))
Data exclusions	No data excluded.
Replication	At least two independent experiments were conducted for all the biochemical and cell-based experiments and similar results were obtained. For ITC experiments, titration was performed twice with similar kinetics for wild-type proteins and once for NLGN3 mutant proteins. Details are given in the "Methods" section.
Randomization	For behavioral experiments, littermate male mice generated by crossing wild-type male mice and heterozygous female mice were genotyped and allocated into experimental and control group. Mice of experimental group and control group were always counterbalanced, as was the position of apparatus and the order of experiment between groups.<br>For other animal experiments, all the male littermates with both wild-type and mutant mice were used.
Blinding	Biochemical, immunohistochemical and electrophysiological analyses of mutant mice were performed in a blind manner with respect to genotype. Data analyses for sniffing behaviors were also carried out by an observer blind to genotype. Quantitative measurements in cocultures from mutant mice were also conducted in a genotype-blind manner. Details are described in the "Methods" section.

Reporting for specific materials, systems and methods

We require information from authors about some types of materials, experimental systems and methods used in many studies. Here, indicate whether each material, system or method listed is relevant to your study. If you are not sure if a list item applies to your research, read the appropriate section before selecting a response.

Materials & experimental systems

n/a	Involved in the study	
[ ]	[x]	Antibodies
[ ]	[x]	Eukaryotic cell lines
[x]	[ ]	Palaeontology and archaeology
[ ]	[x]	Animals and other organisms
[x]	[ ]	Human research participants
[x]	[ ]	Clinical data
[x]	[ ]	Dual use research of concern

Methods

n/a	Involved in the study	
[x]	[ ]	ChIP-seq
[x]	[ ]	Flow cytometry
[x]	[ ]	MRI-based neuroimaging

Antibodies

Antibodies used	WB: mouse anti-NLGN3; BioLegend (MMS-5166-100), 1/200 (S1 fraction) or 1/400 (co-IP sample)<br>WB: mouse anti-Gephyrin; Synaptic Systems (147111), 1/500<br>WB: mouse anti-VAMP2; Synaptic Systems (104211), 1/2000<br>WB: rabbit anti-actin; Santa Cruz Biotechnology (sc-1616-R), 1/1000<br>WB: rabbit anti-PSD95; a gift from Dr. Watanabe, Hokkaido University, 1/600<br>WB: goat anti-VGAT; a gift from Dr. Watanabe, Hokkaido University, 1/600<br>WB: rabbit anti-VGluT1; Operon (custom-made), 1/1000<br>WB: mouse anti-PTPRD; Abcam (ab233806), 1/500<br>WB: mouse anti-His; MBL (M089-3), 1/1000<br>ICC: rabbit anti-Shank2; Frontier Institute (Shank2-Rb-Af750), 1/200<br>ICC: mouse anti-NLGN3; BioLegend (MMS-5166-100), 1/200<br>ICC: mouse anti-Gephyrin; Synaptic Systems (147011), 1/1000<br>IHC: rabbit anti-VGluT1; a gift from Dr. Watanabe, Hokkaido University, 1/600<br>IHC: goat anti-VGAT; a gift from Dr. Watanabe, Hokkaido University, 1/600<br>IP: rabbit anti-NLGN3; Frontier Institute (Nlgn3-Rb-Af1010)<br>IP: rabbit IgG; Rockland (609-4103)
Validation	mouse anti-NLGN3; BioLegend (MMS-5166-100): evaluated for WB using recombinant NLGN3 proteins used in the crystallization in this study<br>mouse anti-Gephyrin; Synaptic Systems (147111): validated for WB using crude synaptic membrane fraction of rat brain (https://sysy.com/product/147111#gallery-1)<br>mouse anti-VAMP2; Synaptic Systems (104211): validated for WB using crude synaptosomal fraction of rat brain (https://sysy.com/product/104211#gallery-1)<br>rabbit anti-actin; Santa Cruz Biotechnology (sc-1616-R): validated for WB using mouse brain extract (Miya, K., et al., 2008. J. Comp. Neurol. 510,641–654)<br>rabbit anti-PSD95; a gift from Dr. Watanabe, Hokkaido University: evaluated for western blotting and immunohistochemistry of mouse brain slices (Fukaya & Watanabe,2000. J. Comp. Neurol. 426,572-586)<br>goat anti-VGAT; a gift from Dr. Watanabe, Hokkaido University: evaluated for immunohistochemistry (Yoshida, T., et al. (2011). J. Neurosci. 31, 13485-13499; Miura et al.(2006). J. Neurochem. 97, 1431-1446.)<br>rabbit anti-VGluT1; Operon (custom-made): evaluated for immunohistochemistry using mouse brain slices and confirmed colocalization with signals of previously characterized goat anti-VGluT1 (Uemura et al., (2007). J. Neurosci. 27,12096-12108)<br>mouse anti-PTPRD; Abcam (ab233806): validated for WB using positive control PTPRD (AA: extra 1077-1265)-hIgGFc transfected HEK-293 cell lysate (Abcam)<br>mouse anti-His; MBL (M089-3): validated for WB using recombinant His- Azami-Green and His-EGFP proteins (https://ruo.mbl.co.jp/bio/dtl/dtlfiles/M089-3-v5.pdf)<br>rabbit anti-Shank2; Frontier Institute (Shank2-Rb-Af750): evaluated for immunohistochemistry using brain slices of wild-type mouse (Frontier Institute, https://nittobo-nmd.co.jp/pdf/reagents/Shank2.pdf)<br>mouse anti-Gephyrin; Synaptic Systems (147011): evaluated for immunohistochemistry and immunocytichemistry (Synaptic Systems, https://www.sysy.com/product/147011)<br>rabbit anti-VGluT1; a gift from Dr. Watanabe, Hokkaido University: evaluated for immunohistochemistry (Yoshida, T., et al. (2011). J. Neurosci. 31, 13485-13499.<br>rabbit anti-NLGN3; Frontier Institute (Nlgn3-Rb-Af1010): validated for IP using P2' fraction of wild-type mouse brain (this study, Fig. 2e)<br>rabbit IgG; Rockland (609-4103): validated as negative control using P2' fraction of wild-type mouse brain (this study, Fig. 2e)

Eukaryotic cell lines

Policy information about cell lines

Cell line source(s)	HEK293T cell line: purchased from Thermo Fisher Scientific<br>FreeStyle 293-F cell line: purchased from Thermo Fisher Scientific
Authentication	Non of the cell lines used were authenticated.
Mycoplasma contamination	Only HEK293T cell line used in the study was tested for mycoplasma and was not contaminated.
Commonly misidentified lines<br>(See ICLAC register)	Commonly misidentified lines were not used in the study.

Animals and other organisms

Policy information about studies involving animals; ARRIVE guidelines recommended for reporting animal research

Laboratory animals	Mice:

	ICR; male and female, P0 (cultured neuronal experiments)<br>C57BL/6N; male, 2-month-old (Real-time PCR)<br>Nlgn3mf on C57BL/6N background; male,<br>P0 (cultured neuronal experiments)<br>P13-16 (electrophysiological experiments)<br>4-6 w (western blotting, co-IP, and immunohistochemistry)<br>adult over 3 months (behavioral testing)<br>Nlgn3hse on C57BL/6N background; male,<br>P0 (cultured neuronal experiments)<br>P13-16 (electrophysiological experiments)<br>4-6 w (western blotting, co-IP, and immunohistochemistry)<br>adult (behavioral testing)<br>Nlgn3 KO on C57BL/6J background; male, P0 (cultured neuronal experiments)<br>Nlgn3R/C on C57BL/6J background; male, P0 (cultured neuronal experiments)
Wild animals	This study did not involve wild animals.
Field-collected samples	This study did not involve field-collected samples.
Ethics oversight	All the animal experiments were approved by the Animal Experiment Committee of the University of Toyama and conducted in accordance with the Guidelines for the Care and Use of Laboratory Animals of the University of Toyama.

Note that full information on the approval of the study protocol must also be provided in the manuscript.
